# Geographic Distribution of Suspected Alpha-gal Syndrome Cases — United States, January 2017–December 2022

**DOI:** 10.15585/mmwr.mm7230a2

**Published:** 2023-07-28

**Authors:** Julie M. Thompson, Ann Carpenter, Gilbert J. Kersh, Tyler Wachs, Scott P. Commins, Johanna S. Salzer

**Affiliations:** ^1^Division of Vector-Borne Diseases, National Center for Emerging and Zoonotic Infectious Diseases, CDC; ^2^Eurofins Viracor, Lenexa, Kansas; ^3^Division of Rheumatology, Allergy, and Immunology, Department of Medicine, School of Medicine, University of North Carolina at Chapel Hill, Chapel Hill, North Carolina.

**Summary**

**What is already known about this topic?**

Alpha-gal syndrome (AGS) is an emerging, tick bite–associated allergic condition characterized by potentially life-threatening hypersensitivity to an oligosaccharide found in most mammalian meat and products derived from it; however, in the absence of national surveillance, the geographic distribution and number of cases are largely unknown.

**What is added by this report?**

The number of suspected AGS cases in the United States has increased substantially since 2010, and states with established populations of lone star ticks are most affected, although suspected AGS cases were also identified in areas outside of this tick’s range.

**What are the implications for public health practice?**

These data can facilitate initiating AGS surveillance, improve health care provider education in high-risk areas, and enhance targeted public health outreach and prevention.

## Abstract

Alpha-gal syndrome (AGS) is an emerging, tick bite–associated allergic condition characterized by a potentially life-threatening immunoglobulin E (IgE)–mediated hypersensitivity to galactose-alpha-1,3-galactose (alpha-gal), an oligosaccharide found in most nonprimate mammalian meat and products derived from these mammals. Specific symptoms and severity of AGS vary among persons, and no treatment or cure is currently available. During 2010–2018, more than 34,000 suspected cases of AGS were identified in the United States, but current knowledge of where cases occur is limited. This study examined alpha-gal–specific IgE (sIgE) antibody testing results submitted to the commercial laboratory responsible for nearly all testing in the United States before 2022 to assess the geographic distribution and magnitude of this emerging condition. During January 1, 2017–December 31, 2022, a total of 357,119 tests were submitted from residences in the United States, corresponding to 295,400 persons. Overall, 90,018 (30.5%) persons received a positive test result in the study period, and the number of persons with positive test results increased from 13,371 in 2017 to 18,885 in 2021. Among 233,521 persons for whom geographic data were available, suspected cases predominantly occurred in counties within the southern, midwestern, and mid-Atlantic U.S. Census Bureau regions. These data highlight the evolving emergence of AGS and can be used to help state and local health agencies initiate surveillance and target public health outreach and health care provider education to high-risk localities.

## Introduction

Alpha-gal syndrome (AGS) is an emerging, tick bite–associated allergic condition characterized by a potentially life-threatening immunoglobulin E (IgE)–mediated hypersensitivity to galactose-alpha-1,3-galactose (alpha-gal), an oligosaccharide found in most nonprimate mammalian tissue and products derived from these mammals, such as milk, other dairy products, and some pharmaceutical products (*1*). Specific signs and symptoms and severity of AGS vary among persons (*2*), and no treatment or cure is currently available (*1*). More than 34,000 suspected AGS cases* were identified in the United States during 2010–2018 (*3*), but knowledge of where cases occurred is limited. This study examined alpha-gal–specific IgE (sIgE) antibody testing results submitted to the commercial laboratory responsible for nearly all testing in the United States before 2022^†^ to describe the geographic distribution and magnitude of this emerging condition in the United States.

## Methods

Deidentified data from sIgE tests^§^ and panels^¶^ submitted in the United States during January 1, 2017–December 31, 2022, were obtained from Eurofins Viracor, the clinical testing laboratory responsible for nearly all testing in the United States before 2022 (www.eurofins-viracor.com), and contained the following variables: patient identification number, age, sex (male, female, or unknown), date of testing, test result provided in kilounits of alpha-gal sIgE per liter (kU/L), and patient state of residence and zip code. No clinical data or travel histories of persons receiving testing were provided. Observations with invalid state entries or entries from outside the United States were excluded. An alpha-gal sIgE test result ≥0.1 kU/L was considered positive. For persons who received one test, a person was suspected to have AGS if they received a positive test, and a person was considered to not have AGS if a negative test was received. For persons who received multiple tests, a person who received at least one positive test result was suspected to have AGS, and a person who received all negative test results was considered to not have AGS. The date and location of residence at the time of the first positive test result (among persons with suspected AGS) or the first negative test result (among those who did not have AGS) were recorded. Means and SDs were calculated for continuous variables, and frequencies and percentages were calculated for categorical and ordinal variables. Risk ratios (RRs), 95% CIs, and p*-*values were calculated to determine associations with positive test results. Pearson’s chi-square tests, Cochran-Armitage test for trend, and student’s *t *tests with unequal variances were used to compare categorical, ordinal, and continuous variables, respectively. All analyses were performed using SAS software (version 9.4; SAS Institute). 

Counties of patient residences were derived from original zip code data.** The number of persons with positive test results per 1 million (1M) population per year (PPY) were calculated for counties using population estimates from the U.S. Census Bureau.^††^ Counties with suspected AGS cases were assigned to one of three equal proportioned categories: low (<11 suspected AGS cases per 1M PPY), medium (11–87), and high (>87). Counties without suspected AGS cases were assigned to a zero category. QGIS (version 3.28.2; QGIS Project) was used for map generation. This activity was reviewed by CDC and was conducted consistent with applicable federal law and CDC policy.^§§^

## Results

During January 1, 2017–December 31, 2022, a total of 357,119 tests were submitted from U.S. residences^¶¶^, corresponding to 295,400 persons who were included in this analysis. Among these, 235,752 (80%) reported state of residence, and 233,521 (79%) reported zip code of residence. The majority of persons who received testing received one test during the study period, but 36,257 persons (12.3%) received more than one test. Overall, 188,532 (63.8%) persons receiving testing were female, but 42% of men received a positive test result, compared with 24% of women (Table). Persons who received a positive test result were significantly older (mean = 48 years; SD = 19.9) than were those who received a negative test result (mean = 41 years; SD = 19.6) (p<0.001); among persons aged ≥70 years, 44.6% received a positive test result.

**TABLE:** Characteristics of persons who received testing for alpha-gal–specific immunoglobulin E — United States, January 1, 2017–December 31, 2022

Characteristic	No. (%)			RR (95% CI)	p-value^§^
	Total (N = 295,400)	Positive test result* (n = 90,018)	Negative test result^†^ (n = 205,382)		
**Age, yrs, mean (SD)**	**43.1 (19.9)**	48.2 (19.9)	40.8 (19.6)	NA	<0.001
**Age group, yrs**					
0–9	**12,332**	2,478 (2.8)	9,854 (4.8)	Ref	<0.001
10–19	**32,421**	8,007 (8.9)	24,414 (11.9)	1.3 (1.2–1.4)	
20–29	**36,852**	7,682 (8.5)	29,170 (14.2)	1.0 (1.0–1.1)	
30–39	**46,520**	10,929 (12.1)	35,591 (17.3)	1.2 (1.2–1.3)	
40–49	**49,297**	13,837 (15.4)	35,460 (17.3)	1.6 (1.4–1.6)	
50–59	**47,975**	17,157 (19.1)	30,818 (15.0)	2.2 (2.1–2.3)	
60–69	**40,690**	16,858 (18.7)	23,832 (11.6)	2.8 (2.7–3.0)	
>70	**29,304**	13,064 (14.5)	16,240 (7.9)	3.2 (3.0–3.4)	
**Sex**					
Female	**188,532**	45,257 (50.3)	143,275 (69.7)	Ref	<0.001
Male	**104,629**	43,874 (48.7)	60,755 (29.6)	2.3 (2.2–2.3)	
Unknown	**2,239**	887 (1.0)	1,352 (0.7)	2.1 (1.9–2.3)	
**Year**					
2017^¶^	**35,869**	13,371 (14.9)	22,498 (11.0)	Ref	<0.001
2018	**43,195**	13,821 (15.4)	29,374 (14.3)	0.8 (0.8–0.8)	
2019	**57,327**	17,372 (19.3)	39,955 (19.5)	0.7 (0.7–0.8)	
2020	**56,726**	16,936 (18.8)	39,790 (19.4)	0.7 (0.7–0.7)	
2021	**66,106**	18,885 (21.0)	47,221 (23.0)	0.7 (0.7–0.7)	
2022	**36,177**	9,633 (10.7)	26,544 (12.9)	0.6 (0.6–0.6)	

**Abbreviations:** IgE = immunoglobulin E; kU = kilounit; NA = not applicable; Ref = referent group; RR = risk ratio.

* At least one alpha-gal–specific IgE test result ≥0.1 kU/L in a patient was considered positive.

^† ^All alpha-gal–specific IgE test results <0.1 kU/L in a patient were considered negative.

^§ ^P*-*values were calculated to identify significant difference in trends (age group or year) or interactions (sex) between those who received positive and negative test results; p<0.05 were considered statistically significant.

^¶ ^Year was abstracted for the date of the first positive test result (for persons who received at least one positive test result) and the first negative test (for persons who only received negative test results).

During the study period, 90,018 (30.5%) persons received a positive test result and were classified as having suspected AGS. Each year during the study, approximately 30,000–70,000 persons received testing, although testing peaked at 66,106 persons in 2021, before other commercial laboratories began providing alpha-gal sIgE testing. The percentage of persons who received a positive test result remained at nearly 30% nationally during the study period, and an increasing number of positive test results were received each year until 2022. Each year, 13,371–18,885 persons received a positive test result (mean = 15,003; SD = 3,385.7).

Test results from 79% of persons with available geographic data were used for map generation. The highest numbers of suspected AGS cases were identified in counties within New York (Suffolk [3,746]) and Virginia (Bedford [1,511]); 4% of all suspected cases nationwide resided in Suffolk County, New York. The highest number of suspected AGS cases per 1M PPY were in counties in Virginia (Charlotte [12,273]) and Kentucky (Muhlenberg [6,107]). The highest prevalences of suspected cases (per 1M PPY) were found throughout a nearly contiguous region of the southern, midwestern, and mid-Atlantic United States, particularly parts of Oklahoma, Kansas, Arkansas, Missouri, Mississippi, Tennessee, Kentucky, Illinois, Indiana, North Carolina, Virginia, Maryland, and Delaware (Figure). Counties with moderate and high numbers of suspected cases per 1M PPY were detected in Minnesota and Wisconsin, corresponding to 238 total suspected cases (238 of 2,456 persons tested; 9.7%) during the 6-year study period, and were distinct from this contiguous region. Suspected AGS cases were predominantly located in areas where the lone star tick (*Amblyomma americanum*) is known to be established or reported.

**FIGURE:**
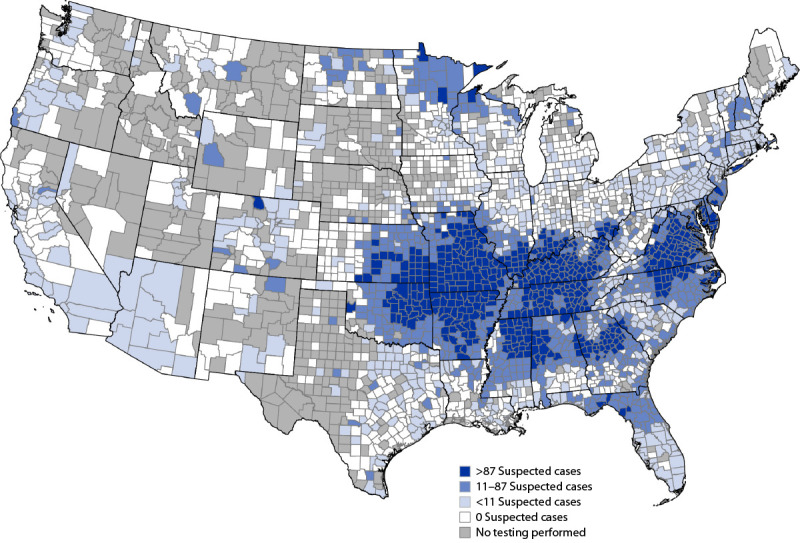
Geographic distribution of suspected alpha-gal syndrome cases* per 1 million population per year — United States, 2017–2022 **Abbreviations:** IgE = immunoglobulin E; IU = international unit; kU = kilounit. * A suspected case of alpha-gal syndrome was defined as being in a person who had confirmatory laboratory evidence (serum or plasma alpha-gal–specific IgE ≥0.1 IU/mL or ≥0.1 kU/L) with no clinical information available.

## Discussion

During 2017–2021, there was an annual increase in positive test results for AGS in the United States. More than 90,000 suspected AGS cases were identified during the study period, and the number of new suspected cases increased by approximately 15,000 each year during the study.

Health care providers (HCPs) in the United States have low awareness of AGS. Among surveyed providers, 42% had never heard of AGS, and 35% reported they were “not too confident” in their ability to diagnose AGS or to manage patients with AGS (*4*). In this study, it was presumed that HCPs submitting alpha-gal sIgE tests had a reasonably high index of clinical suspicion of AGS. Alpha-gal sIgE testing conducted by HCPs with knowledge of AGS and with a high index of suspicion has been shown to have 98% sensitivity (*5*,*6*) and 92% specificity (*6*). Because no clinical data were available in the current study to correlate positive test results with the presence of clinical symptoms consistent with AGS, all cases were considered suspected. However, recent unpublished surveillance data that examined positive alpha-gal sIgE test results at commercial laboratories showed that approximately 90% of persons with a positive test result did have clinical symptoms consistent with AGS (K Cervantes, New Jersey Department of Health, personal communication, July 2023) and that they were classified as having confirmed AGS.

Persons with suspected AGS were predominantly located in areas where the lone star tick is known to be established or reported, particularly throughout Arkansas, Kentucky, Missouri, and Suffolk County, New York. The geographic distribution of AGS is very similar to that of ehrlichiosis, caused by *Ehrlichia chaffeensis* and *E. ewingii*, disease agents also known to be transmitted by the lone star tick. These data therefore support the association previously observed between lone star ticks and alpha-gal sensitization among patients in the United States. This study also identified focal clusters of cases in areas where there are no known established populations of lone star ticks, such as Minnesota and Wisconsin, although these data are relatively sparse, and more information is needed to validate these as cases acquired in those areas. A small retrospective review in Iowa, Minnesota, and Wisconsin found that of 47 AGS patients who received positive alpha-gal sIgE test results, 11 (23%) lived in areas where the lone star tick was not previously known to be present, and some persons reported bites from blacklegged ticks (four; 9%) or lone star ticks (three; 6%), although when these bites occurred relative to symptom onset or how the ticks were identified is not described (*7*). Nevertheless, alpha-gal has been identified in the saliva of other tick species (*8*,*9*), and bites from other tick species are associated with AGS in other parts of the world (*8*). In this investigation, the geography suggests that lone star ticks remain the primary species associated with AGS in the United States, and cases outside the established range of this tick species need to be further investigated to better understand exposure history and contributing factors associated with the onset of this allergic condition.

The results of the current study can aid in initiating national surveillance efforts for this emerging allergic condition and for geographically targeting high-risk populations for public health outreach and HCP education. Whether the increasing numbers of suspected AGS cases seen in this study are an indication of increased awareness, increasing emergence, or both remains unclear. Further, these results support including AGS in community outreach regarding tickborne disease prevention efforts, especially because the health consequences of tick exposures leading to AGS could ultimately be lifelong.

### Limitations

The findings in this report are subject to at least four limitations. First, it is known that other specialty laboratories within academic institutions and allergy clinics have conducted alpha-gal sIgE testing before 2022. In addition, other commercial laboratories have conducted testing since August 2021, which are not reflected in these results and likely contributes to the decrease in suspected AGS cases in 2022. Thus, these results almost certainly underestimate the number of persons seeking testing and persons receiving positive test results. Second, localities associated with patient test results do not necessarily reflect the geographic area where the tick bites or first onset of AGS symptoms occurred, and travel-associated cases certainly are possible. Third, test specificity for AGS is 92% among symptomatic persons (*6*), and false positives are possible. Finally, some of the original data were excluded from the study because of invalid state entries or entries from outside the United States, though this represented <1% of the total sample.

### Implications for Public Health Practice

Because numerous barriers affect access to testing, these test results do not equitably reflect the U.S. populations affected by AGS. Studies have documented that most patients seeking and receiving sIgE testing were more likely to report being non-Hispanic White, with higher incomes, and higher educational attainment (*5*,*10*). The need for repeated clinical visits and access to specialized practitioners, which might span several years before a diagnosis is made (*10*), also creates a testing barrier for patients. These known challenges are likely the reason that only a portion of persons with AGS are tested for alpha-gal sIgE antibodies. The suspected health equity gaps associated with AGS warrant further examination.

AGS is a growing clinical and public health concern for persons in the United States, yet in the absence of a national surveillance system, the prevalence of this condition is largely unknown. More than 34,000 suspected AGS cases were previously identified during 2010–2018, and 20,211 of these were identified during 2010–2016 from alpha-gal sIgE test results (*3*). Together with suspected AGS cases identified from alpha-gal sIgE tests and panels in this study, a total of 110,229 suspected cases were documented during 2010–2022. Assuming 70%–90% of these suspected cases (77,161–99,207) are clinically compatible AGS cases, and assuming that 22%–80% of all persons with AGS have access to knowledgeable HCPs who submit a specimen for alpha-gal sIgE testing, 96,000–450,000 persons in the United States might have been affected by AGS since 2010. A recent survey (*4*) found that approximately 22% of HCPs in the United States were somewhat or very confident that they would be able to diagnose or manage patients with AGS. However, it has been estimated that approximately 80% of AGS patients at specialty clinics received alpha-gal sIgE testing as part of their clinical diagnosis. If testing trends continue, and the geographic range of the lone star tick continues to expand, the number of AGS cases in the United States is predicted to increase during the coming years, presenting a critical need for synergistic public health activities including 1) community education targeting tick bite prevention to reduce the risk for acquiring AGS, 2) HCP education to improve timely diagnosis and management, and 3) improved surveillance to aid public health decision-making.

## Footnotes

* The national standardized case definition accepted in 2021 by the Council of State and Territorial Epidemiologist defined a confirmed case of AGS as being in a person who met the clinical criteria and had confirmatory laboratory evidence (serum or plasma sIgE to alpha-gal ≥0.1 international unit per milliliter or ≥0.1 kU/L). A suspected case of AGS was defined as being in a person who had confirmatory laboratory evidence with no clinical information available. https://ndc.services.cdc.gov/case-definitions/alpha-gal-syndrome-ags/

† Until August 2021, Eurofins Viracor was the primary commercial laboratory offering alpha-gal sIgE testing. Few academic institutions and specialty allergy clinics offered testing before August 2021. Several commercial laboratories began offering alpha-gal sIgE testing in August 2021.

§ Alpha-gal sIgE tests (Eurofins Viracor testing code 30039).

¶ Alpha-gal panels included alpha-gal IgE, beef IgE, pork IgE, and lamb or mutton IgE (Eurofins Viracor testing code 403196P).

** For zip codes that crossed county boundaries, the county with the greatest proportion of residential addresses was selected according to the U.S. Department of Housing and Urban Development’s Office of Policy Development and Research.

†† https://data.census.gov/table?q%20=%20b01003&g%20=%20010XX00US$0500000,&tid%20=%20ACSDT1Y2021.B01003 (Accessed December 1, 2022).

§§ 45 C.F.R. part 46, 21 C.F.R. part 56; 42 U.S.C. Sect. 241(d); 5 U.S.C. Sect. 552a; 44 U.S.C. Sect. 3501 et seq.

¶¶ Test results from 10 specimens were excluded from the analysis. These reported invalid state entries or were reported from outside the United States.
